# Distribution pattern of antibiotic resistance genes in *Escherichia coli* isolated from colibacillosis cases in broiler farms of Egypt

**DOI:** 10.14202/vetworld.2023.1-11

**Published:** 2023-01-03

**Authors:** Mona A. A. Abdel-Rahman, Engy A. Hamed, May F. Abdelaty, Hend K. Sorour, Heba Badr, Wafaa M. Hassan, Azhar G. Shalaby, Ahmed Abd-Elhalem Mohamed, Mohamed A. Soliman, Heba Roshdy

**Affiliations:** Reference Laboratory for Veterinary Quality Control on Poultry Production, Animal Health Research Institute, Agricultural Research Center, Nadi El-Seid Street, Dokki P. O. Box 246, Giza 12618, Egypt

**Keywords:** antibiotic resistance, Egypt, *Escherichia coli*, integron, *mcr*-1, poultry

## Abstract

**Background and Aim:**

Multidrug resistance (MDR) of *Escherichia coli* has become an increasing concern in poultry farming worldwide. However, *E. coli* can accumulate resistance genes through gene transfer. The most problematic resistance mechanism in *E. coli* is the acquisition of genes encoding broad-spectrum β-lactamases, known as extended-spectrum β-lactamases, that confer resistance to broad-spectrum cephalosporins. Plasmid-mediated quinolone resistance genes (conferring resistance to quinolones) and *mcr-1* genes (conferring resistance to colistin) also contribute to antimicrobial resistance. This study aimed to investigate the prevalence of antimicrobial susceptibility and to detect β-lactamase and colistin resistance genes of *E. coli* isolated from broiler farms in Egypt.

**Materials and Methods:**

Samples from 938 broiler farms were bacteriologically examined for *E. coli* isolation. The antimicrobial resistance profile was evaluated using disk diffusion, and several resistance genes were investigated through polymerase chain reaction amplification.

**Results:**

*Escherichia coli* was isolated and identified from 675/938 farms (72%) from the pooled internal organs (liver, heart, lung, spleen, and yolk) of broilers. *Escherichia coli* isolates from the most recent 3 years (2018–2020) were serotyped into 13 serotypes; the most prevalent serotype was O125 (n = 8). The highest phenotypic antibiotic resistance profiles during this period were against ampicillin, penicillin, tetracycline, and nalidixic acid. *Escherichia coli* was sensitive to clinically relevant antibiotics. Twenty-eight selected isolates from the most recent 3 years (2018–2020) were found to have MDR, where the prevalence of the antibiotic resistance genes *ctx*, *tem*, and *shv* was 46% and that of *mcr-1* was 64%. Integrons were found in 93% of the isolates.

**Conclusion:**

The study showed a high prevalence of *E. coli* infection in broiler farms associated with MDR, which has a high public health significance because of its zoonotic relevance. These results strengthen the application of continuous surveillance programs.

## Introduction

Avian colibacillosis is a major poultry disease that affects all ages of poultry globally and is caused by *Escherichia coli*. The disease is associated with septicemia, pericarditis, airsacculitis, perihepatitis, peritonitis, and other extraintestinal lesions in poultry and with high economic loss due to the high mortality and low productivity of poultry farms. Many studies have reported an intensive increase in multidrug resistance (MDR) in *E. coli* strains [1–4].

Antibiotics are used for treatment and prophylaxis against bacterial infections as well as growth promoters, particularly in chicken production; most antibiotics used in veterinary practices are very similar to those used for the clinical treatment of human diseases [5]. During the past decade, antibiotic resistance that emerged as a global problem has engaged international health agencies to comply with the management policies for antibiotic use to avoid exacerbating the problem and to ensure the protection of public health [6–8]. Foodborne bacteria may carry and transfer resistance genes to humans [9]. The resistant bacteria acting as a reservoir could then transfer those genes to commensal microorganisms in addition to pathogenic microorganisms inside the human digestive tract [10]. Transfer of MDR in this manner would make it very difficult to treat bacterial infections [11].

β-lactam antibiotics are some of the most widely used antibiotics that produce an increase in antibiotic-resistant isolates because of increased selective pressure [12]. β-lactamases that hydrolyze an expanded spectrum of cephalosporins and monobactams are classified as extended-spectrum β-lactamases (ESBLs) [13].

Extended-spectrum β-lactamases of Class A mainly includes a variety of hydrolyzing enzymes, such as TEM, SHV, CTX-M, and VEB, and the highest number of variants is found in the CTX-M enzymes. Studies over the past 10 years have revealed that the most widely used CTX-M β-lactamase-producing bacteria were *Enterobacteriaceae* [14]. There are two classifications of β-lactamases that are currently in use. One is based on the amino acid sequence that includes a serine utilized for lactate hydrolysis. These enzymes are subdivided into Classes A, C, and D enzymes and Class B, which respond to metalloenzymes that require divalent zinc ions for substrate hydrolysis. The updated classification is Group 1, including Class C; Group 2, including Class Al; and Group 3, including Group B [13, 15]. *Escherichia coli* isolated from broiler farms have been shown to be resistant to penicillins and cephalosporins as well as aztreonam mainly due to the production of CTX-M, TEM, and SHV β-lactamases which are encoded by the *bla*CTX-M, *bla*SHV, and *bla*TEM genes, respectively [16].

Quinolone is considered one of the important antibiotics used in treating *E. coli* infections in poultry farms. The presence of quinolone resistance genes in bacteria is an evolving problem in *E. coli* infection control. Quinolones work by interfering with gyrase and topoisomerase IV activity, leading to fragmentation of the bacterial chromosome; this subsequently drives mutations in the gyrase and topoisomerase IV genes and the development of bacterial resistance to quinolones [17, 18].

Colistin resistance is encoded by the *mcr*-1 gene, which has been detected in isolates of *Enterobacteriaceae* from humans, food, and livestock [19–21]. Colistin resistance usually develops by mutations in the lipid synthesis enzymes of the bacterial outer membrane [22–24]. Recently, colistin has been widely used as the drug of choice for several bacterial infections, especially in cases infected by MDR Gram-negative bacteria, especially β-lactamase-resistant *Enterobacteriaceae* [19, 25]. The extensive usage of colistin in the animal production industry as a tool for productivity improvement, besides infection control, contributes to the appearance of colistin resistance in *E*. *coli*, which is usually accompanied by the emergence of the plasmid-mediated colistin resistance determinants, *mcr*-1, *mcr*-2, *mcr*-3, *mcr*-4, and *mcr*-5 [26].

Integrons are bacterial genetic elements that are commonly distributed between Gram-negative bacteria in humans and animals. Furthermore, they can be encoded with antimicrobial resistance factors and are subsequently known as resistance integrons or MDR integrons [27]. This class of integrons is usually detected in clinical isolates and is known as clinical integrons [28] and acts as a genetic construction kit for bacteria [29, 30]. Furthermore, integrons are involved in developing and disseminating antibiotic resistance genes in enteric bacteria.

There are several virulence factors detected in *E. coli* strains that have been isolated from cellulitis and other colibacillosis lesions [31, 32]. Shiga toxins (*Stx*) are the main virulence factors that are responsible for *E. coli* pathogenicity, and these occur as two genes: *stx1* and *stx2* [33]. In addition, the increased serum survival (*iss*) gene found on episomes can control expression of protectins/serum resistance genes to enhance the ability of bacteria to survive in the host serum [34].

This study aimed to determine the antimicrobial susceptibility of *E. coli* isolated from broiler farms in different localities in Egypt and assess the degree of antimicrobial resistance, such as the presence of β-lactamase and colistin resistance genes.

## Materials and Methods

### Ethical approval

The study procedures were approved by the Animal Care Committee of the Animal Health Research Institute (AHRI) Dokki, Giza, Egypt under protocol number (AHRI-42429/2020).

### Study period and location

This study was conducted from January 2014 to December 2020 in Reference Laboratory for Veterinary Quality Control on Poultry production - Animal Health Research Institute, Egypt.

### Samples

This study was conducted to trace *E. coli* isolation. Samples were taken from 938 broiler poultry farms located in 25 governorates in Egypt; the number of examined farms from each governorate differs according to the local distribution of poultry farms in Egypt. From each farm, five clinically diseased birds from 7 to 35 days of age were inspected postmortem; bacteriological examination was conducted on the collected pooled organs (liver, lung, spleen, heart, and yolk) from diseased birds to represent one sample. The diseased birds showed different rates of mortalities, diarrhea, colisepticemia, airsacculitis, perihepatitis, and pericarditis.

### Isolation and identification of *E. coli*

*Escherichia coli* was isolated and identified according to Nolan *et al*. [1]. Briefly, all the collected samples were pre-enriched in buffered peptone water (Lab M, UK) and incubated aerobically at 37°C for 24 h. A loopful of the broth culture was inoculated onto MacConkey agar (Neogen, US) and eosin methylene blue agar (Lab M) plates, which were incubated at 37°C for 24 h. The isolated colonies were identified morphologically and biochemically (oxidase strips and triple sugar iron agar were from Oxoid, UK; urea, Simmons’ citrate agar, and peptone water were from Lab M; and Kovacs reagent was from HiMedia, India) [1]. In addition, antisera against somatic (O) antigens (Denka Seiken Co., Tokyo, Japan) were used for serotyping isolated *E. coli* following the manufacturer’s instructions.

### Antimicrobial sensitivity test (AST)

An AST was conducted for all isolates using a disk diffusion test, as previously described [35] against 18 antibiotics (HiMedia^®^), which were amoxicillin-clavulanate (AMC, 30 μg), ampicillin (AMP, 10 μg), ciprofloxacin (CIP, 5 μg), cefotaxime (CTX, 30 μg), chloramphenicol (C, 30 μg), danofloxacin (DFX 5 μg), doxycycline (DOX 30, μg), fosfomycin (FOS 200 μg), levofloxacin (LEV, 5 μg), Penicillin (P 10 μg), enrofloxacin (ENR 5 μg), colistin sulfate (CT, 10–25 μg), imipenem (IMP, 10 μg), nalidixic acid (NA, 30 μg), norfloxacin (NX, 10 μg), streptomycin (S, 10 μg), sulfamethoxazole-trimethoprim (SXT, 25 μg), and tetracycline (T, 30 μg). The results were and interpreted according to CLSI [36]. The susceptibility of *E. coli* isolates to individual antimicrobial agents was determined and interpreted following aerobic incubation at 37°C for 18–24 h, according to the Clinical and Laboratory Standard Institute guidelines [36]. The antimicrobial susceptibility of colistin was determined using disk diffusion susceptibility testing using colistin disks (Oxoid) containing 10 μg of antibiotic. The disk zone diameters were interpreted according to a previous report [37]. Resistant and intermediately resistant isolates were collectively referred to as non-susceptible, as previously described [38]. Isolates were considered to be MDR strains when found to be non-susceptible to at least one agent in three or more antimicrobial different classes of antimicrobial agents.

### Molecular assessment

The 28 selected *E. coli* isolates from 2018 to 2020 were further tested using polymerase chain reaction (PCR) for the presence of *bla*TEM, *bla*SHV, *bla*CTX-M, *mcr*-1, *qnrA*, *qnrB*, *papC*, *integron*, and *iss* genes.

DNA was extracted from culture broth using a QIAamp DNA Mini Kit (Qiagen, Germany, GmbH Catalogue No. 51304). The extracted DNA was used in subsequent PCR assays for species confirmation and to detect genes responsible for virulence and antimicrobial agent resistance. The polymerase chain reaction was performed in a final volume of 25 μL that contained 12.5 μL of EmeraldAmp MAX PCR Master Mix (EmeraldAmp GT [2× premix], Japan), 1 μL of each primer (20 pmol), 4.5 μL of diethyl pyrocarbonate water, and 6 μL of the DNA template. The reaction was performed in a Biometra thermal cycler, T3000 (Germany). The oligonucleotide primers (Table-1) [39–45] were supplied by Metabion, Germany.

**Table-1:** Primers used for antibiotic resistance genes and virulence detection.

Primer	Sequence	Amplicon size	Reference

	(5’-3’)		
Beta-lactams			
*bla*SHV	AGGATTGACTGCCTTTTTG	392 bp	[39]
	ATTTGCTGATTTCGCTCG		
*bla*TEM	ATCAGCAATAAACCAGC	516 bp	
	CCCCGAAGAACGTTTTC		
*bla*CTX-M	ATG TGC AGY ACC AGT AAR GTK ATG GC	593 bp	[40]
	TGG GTR AAR TAR GTS ACC AGA AYC AGC GG
Colistin resistance gene			
*Mcr1*	CGGT CAGTCCGTTTGTTC	308 bp	[41]
	CTTGGTCGGTCTGTAGGG
Quinolone resistance			
*qnrA*	ATTTCTCACGCCAGGATTTG	516 bp	[42]
	GATCGGCAAAGGTTAGGTCA		
*qnrB*	GATCGTGAAAGCCAGAAAGG	469 bp	
	ACGATGCCTGGTAGTTGTCC
Integron			
*hep*	TGCGGGTYAARGATBTKGATTT	491 bp	[43]
	CARCACATGCGTRTARAT
Virulence genes			
*papC*	TGTATCACGCAGTCAGTAGC	501 bp	[44]
	CCGGCCATATTCACATAA		
*ISS*	ATGTTATTTTCTGCCGCTCTG	266 bp	[45]
	CTATTGTGAGCAATATACCC		

Polymerase chain reaction products were separated by electrophoresis [46] on a 1% agarose gel (AppliChem, Germany, GmbH) in 1× TBE buffer at room temperature (23°C to 27°C) using a gradient of 5 V/cm. Each well was loaded with 15 μL of the PCR product. A GelPilot 100 bp (Qiagen) ladder was used to determine the fragment sizes. The gel was photographed using a gel documentation system (Biometra BDA digital, Germany), and the data were analyzed using gel documentation (Alpha Innotech, Biometra, Germany) and specific software (automatic image capture software, Protein Simple, formerly Cell Bioscience, USA). The amplification conditions of the primers during PCR are shown in Table-2.

**Table-2:** Cycling conditions of the primers during PCR.

Gene	Primary denaturation	Secondary denaturation	Annealing	Extension	No. of cycles	Final extension
Beta-lactams						
*bla*SHV	94°C 5 min	94°C 30 s	54°C 40 s	72°C 40 s	35	72°C 10 min
*bla*CTX-m *bla*TEM	94°C 5 min	94°C 30 s	54°C 40 s	72°C 45 s	35	72°C 10 min
Colistin resistance gene						
*Mcr1*	94°C 5 min	94°C 30 s	55°C 40 s	72°C 45 s	35	72°C 10 min
Quinolone resistance						
QnrA	94°C 5 min	94°C 30 s	55°C 45 s	72°C 45 s	35	72°C 10 min
QnrB	94°C 5 min	94°C 30 s	55°C 45 s	72°C 45 s	35	72°C 10 min
Integron						
*hep*	94°C 5 min	94°C 30 s	55°C 40 s	72°C 45 s	35	72°C 10 min
Virulence genes						
papC	94°C 5 min	94°C 30 s	58°C 40 s	72°C 45 s	35	72°C 10 min
*Iss*	94°C 5 min	94°C 30 s	54°C 30 s	72°C 30 s	35	72°C 10 min

PCR=Polymerase chain reaction

The amplification efficiency was verified for positive field samples that may contain the tested genes, which were previously examined in a Veterinary Quality Control Reference Laboratory for Poultry Production, Animal Health Research Institute, Egypt.

## Results

### *Escherichia coli* isolation, identification, and serotyping

**Figure-1:**
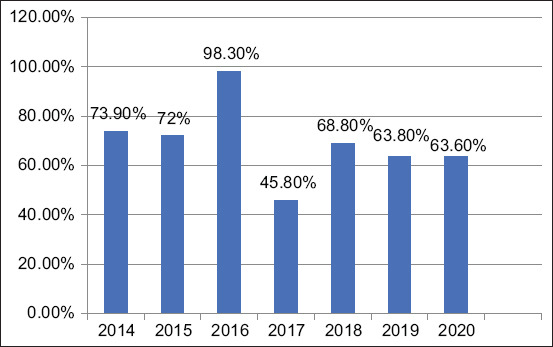
Prevalence of *Escherichia coli* isolated from 2014 to 2020.

*Escherichia coli* was isolated from 675/938 (72%) examined poultry farms between 2014 and 2020. The highest prevalence of *E. coli* was 98.3% in 2016 and the lowest prevalence of *E. coli* was 45.8% in 2017, while in other years, the prevalence ranged from 63.6% to 73.9%, as shown in Table-3 and Figure-1. The highest prevalence of *E. coli* among poultry farms in Egyptian governorates was 98.3% in 2016 (175/178), as shown in Table-4.

**Table-3:** Prevalence of *E. coli* isolated from poultry farms in period (2014–2020).

Year/No. of examined samples	*E. coli*	

	No. of positive samples	%
2014 (n = 134)	99	73.9
2015 (n = 118)	85	72
2016 (n = 178)	175	98.3
2017 (n = 83)	38	45.8
2018 (n = 144)	99	68.8
2019 (n = 138)	88	63.8
2020 (n = 143)	91	63.6
Total (n = 938)	675	72

*E. coli=Escherichia coli*

**Table-4:** Summary about investigated poultry farms in different governorates.

Governorate	2016		2017		2018		2019		2020	
				
	Tested farms	Positive farms	Tested farms	Positive farms	Tested farms	Positive farms	Tested farms	Positive farms	Tested farms	Positive farms
Al Bahr Al Ahmar	1	1	2	0	1	1	NA	NA	NA	NA
Al Beheira	55	55	7	2	61	57	4	2	4	2
Al Dakahlia	13	12	10	7	16	9	34	27	34	27
Al Fayoum	4	3	2	2	NA	NA	NA	NA	4	2
Al Gharbia	1	1	4	4	3	2	NA	NA	NA	NA
Al Giza	9	6	13	7	9	4	56	29	56	29
Al Minia	2	2	NA	NA	NA	NA	NA	NA	NA	NA
Al Monofiya	1	1	10	3	NA	NA	5	2	5	2
Al Qahera	14	8	1	1	4	3	9	5	9	5
Al Qalyubia	7	4	9	4	2	1	6	4	6	4
Al Sharqiya	6	6	10	1	18	0	6	4	6	4
Alexandria	3	3	2	1	4	2	9	8	9	8
Beni Suef	2	2	4	1	1	0	NA	NA	NA	NA
Ismailia	23	19	4	1	NA	NA	7	6	7	6
Luxor	26	25	1	1	8	7	NA	NA	NA	NA
Qena	6	6	1	1	13	12	NA	NA	NA	NA
Kafer El Sheikh	2	2	NA	NA	NA	NA	1	0	1	0
Suez	NA	NA	NA	NA	NA	NA	NA	NA	NA	NA
Mersa Matruh	NA	NA	NA	NA	1	0	NA	NA	NA	NA
North Sinai	1	1	NA	NA	NA	NA	1	1	1	1
South Sinai	NA	NA	1	0	NA	NA	NA	NA	NA	NA
Port Said	NA	NA	NA	NA	NA	NA	NA	NA	NA	NA
Aswan	NA	NA	1	1	1	1	NA	NA	NA	NA
Sohag	NA	NA	1	1	2	0	NA	NA	1	1
Asyut	NA	NA	NA	NA	NA	NA	NA	NA	NA	NA
Total	178	175	83	38	144	99	138	88	143	91
Positivity rate	98.3%		45.8%		68.8%		63.8%		63.6%	

NA=Not applicable , *E. coli=Escherichia coli*

The selected *E coli* isolates from the most recent years (2018–2020) were serotyped into 13 different serotypes. The isolate with the highest prevalence was O125 (n = 8) followed by 0111 (n = 5) and then the remaining serotypes: O55 (n = 3), O15 (n = 2), O157 (n = 2), O 55 (n = 1), O6 (n = 1), O151 (n = 1), O 127 (n = 1), O166 (n = 1), O 143 (n = 1), O86 (n = 1), and O151 (n = 1).

### Antimicrobial susceptibility patterns of the isolated *E. coli*

**Figure-2:**
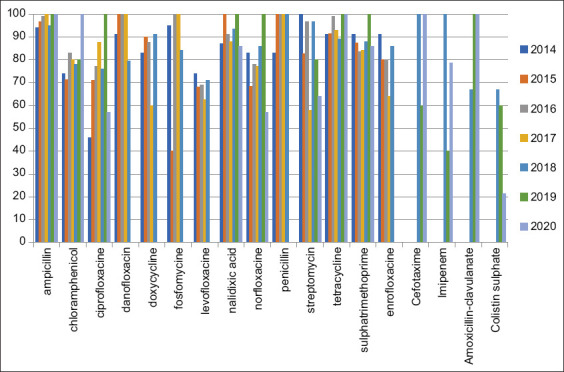
Antibiotics resistance profile of *Escherichia coli* isolated from 2014 to 2020.

*Escherichia coli* isolates were tested for their susceptibility using the disk diffusion technique against 18 antibiotics: AMP, C, CIP, DFX, DOX, FOS, LEV, NA, NX, P, S, T, trimethoprim, ENR, CTX, IMP, AMC + clav, and CT. Most *E. coli* isolates showed the highest resistance percentage to AMP, P, T, and NA, while the lowest resistance percentage (49%) was shown with CT. Resistance to other antibiotics ranged from 88.5% to 69.7% during the period from 2014 to 2020, as shown in Table-5 and Figure-2. These results show that all *E. coli* isolates were considered as MDR strains (Table-6).

**Table-5:** Antibiotics resistance profile of *E. coli* isolates from 2014 to 2020.

Antibiotics	2014 (n = 99)	2015 (n = 85)	2016 (n = 175)	2017 (n = 38)	2018 (n = 99)	2019 (n = 88)	2020 (n = 91)	Total (n = 496)
Ampicillin	94%	96.5%	99%	100%	95%	100	100	97.8%
Chloramphenicol	74%	71.4%	83%	80%	78%	80	100	80.9%
Ciprofloxacin	46%	71%	77%	87.5%	76%	100	57	73.5%
Danofloxacin	91%	100%	100%	100%	79.5%	NA	NA	84.3%
Doxycycline	83%	90%	87.5%	60%	91%	NA	NA	85.5%
Fosfomycin	95%	40%	100%	100%	84%	NA	NA	85.5%
Levofloxacin	74%	68%	69%	62.5%	71%	NA	NA	69.7%
Nalidixic acid	87%	100%	91%	88%	93.5%	100	86	92.2%
Norfloxacin	83%	68.5%	78%	77%	86%	100	57	73.2%
Penicillin	83%	100%	100%	100%	100%	NA	NA	95%
Streptomycin	100%	82.8%	96.5%	58%	96.5%	80	64	82.5%
Tetracycline	91%	91.4%	99%	93%	89%	100	100	94.8%
Sulfamethoxazole-trimethoprim	91%	87.2%	83.5%	84%	88%	100	86	88.5%
Enrofloxacin	91%	80%	80%	64%	86%	NA	NA	82.4%
Cefotaxime	NA	NA	NA	NA	100%	60%	100%	86.7%
Imipenem	NA	NA	NA	NA	100%	40%	78.5%	72.8%
Amoxicillin - clavulanate	NA	NA	NA	NA	67%	100%	100%	89%
Colistin sulfate	NA	NA	NA	NA	67%	60%	21.5%	49.3%

NA=Not applicable, *E. coli=Escherichia coli*

**Table-6:** Phenotypic resistance, resistance determinants and virulence genes found in *E. coli* isolates.

No. of strain	Serotype	Year	Phenotypic antibiotic resistance	Resistance genes identified	Virulence genes
1	O6	2018	AMC^30^, AMP^10^, CTX^30^, CIP^5^, NX^10^, NA^10^, TE^30^, SXT	*CTX, TEM,* *mcr*-1, *hep*	*ISS*
2	O151	2018	AMP^10^, IMP^10^, NA^10^, TE^30^, SXT	*TEM, hep*	*ISS, papC*
3	O143	2018	AMP^10^, IMP^10^, NA^10^, TE^30^, SXT	*TEM, mcr*-1, *hep*	*ISS*
4	O125	2018	AMP^10^, CTX^30^, IMP^10^, NA^10^, TE^30^, SXT	*TEM, SHV, hep*	*ISS*
5	O151	2018	AMP^10^, IMP^10^, NA^10^, TE^30^, SXT	*TEM*	*ISS papC*
6	O15	2018	AMC^30^, AMP^10^, CTX^30^, IMP^10^, C^30^, CIP^5^, NX^10^, NA^10^, TE^30^, SXT, S^10^	*CTX, TEM,* *Qrna, hep*	*ISS papC*
7	O125	2018	AMC^30^, AMP^10^, CTX^30^, IMP^10^, C^30^, CIP^5^, NX^10^, NA^10^, TE^30^, SXT, S^10^	*CTX, TEM,* *mcr*-1, *hep*	*ISS*
8	O15	2018	AMC^30^, AMP^10^, CTX^30^, IMP^10^, C^30^, CIP^5^, NA^10^, TE^30^, SXT, S^10^	*CTX, TEM,* *Qrna, hep*	*ISS, papC*
9	O 125	2018	AMP^10^, CTX^30^, IMP^10^, NA^10^, TE^30^, SXT, S^10^	*TEM, hep*	*ISS, papC*
10	O125	2019	AMC^30^, AMP^10^, CTX^30^, IMP^10^, C^30^, CIP^5^, NX^10^, NA^10^, TE^30^, SXT, S^10^	*TEM, hep*	*ISS*
11	O125	2019	AMC^30^, AMP^10^, CTX^30^, C^30^, CIP^5^, NX^10^, NA^10^, TE^30^, SXT, S^10^	*CTX, TEM, SHV, MCR*-1, *hep*	*ISS, papC*
12	O125	2019	AMC^30^, AMP^10^, CTX^30^, C^30^, CIP^5^, NA^10^, TE^30^, SXT, S^10^	*TEM, MCR*-1, *hep*	*ISS, papC*
13	O125	2019	AMC^30^, AMP^10^, IMP^10^, C^30^, CIP^5^, NX^10^, NA^10^, TE^30^, SXT, S^10^	*TEM, MCR*-1, *hep*	*ISS, papC*
14	O 55	2019	AMC^30^, AMP^10^, CTX^30^, CIP^5^, NX^10^, NA^10^, TE^30^, SXT	*TEM, MCR*-1, *hep*	*ISS*
15	O125	2020	AMC^30^, AMP^10^, CTX^30^, C^30^, CIP^5^, NX^10^, NA^10^, TE^30^, SXT	*CTX, TEM,* *MCR*-1, *hep*	*ISS*
16	O125	2020	AMC^30^, AMP^10^, C^30^, TE^30^, S^10^	*MCR-1*	*ISS*
17	O111	2020	AMC^30^, AMP^10^, CTX^30^, C^30^, CT, CIP^5^, NX^10^, NA^10^, TE^30^, SXT	*CTX, TEM, SHV, MCR*-1, *hep*	*ISS*
18	O111	2020	AMC^30^, AMP^10^, CTX^30^, NX^10^, NA^10^, TE^30^, SXT, S^10^	*CTX, TEM, SHV, MCR*-1, *hep*	*ISS*
19	O111	2020	AMC^30^, AMP^10^, CTX^30^, C^30^, NA^10^, TE^30^, S^10^	*CTX, TEM,* *MCR*-1, *hep*	*ISS*
20	O111	2020	AMC^30^, AMP^10^, CTX^30^, C^30^, NA^10^, TE^30^, SXT, S^10^	*CTX, TEM,* *MCR*-1, *hep*	*ISS*
21	O111	2020	AMC^30^, AMP^10^, CTX^30^, C^30^, NA^10^, TE^30^, SXT, S^10^	*CTX, TEM, SHV, hep*	
22	O127	2020	AMC^30^, AMP^10^, CTX^30^, IMP^10^, C^30^, NA^10^, TE^30^, SXT, S^10^	*CTX, TEM, SHV, hep*	
23	O157	2020	AMC^30^, AMP^10^, CTX^30^, IMP^10^, C^30^, NA^10^, TE^30^, S^10^	*TEM, SHV,* *MCR*-1, *hep*	*ISS*
24	O55	2020	AMC^30^, C^30^, NA^10^, TE^30^, SXT, S^10^	*hep*	*ISS, PAPC*
25	O166	2020	AMC^30^, AMP^10^, CTX^30^, NA^10^, SXT	*TEM, SHV, MCR*-1, *hep*	*ISS*
26	O86	2020	AMC^30^, AMP^10^, CTX^30^, IMP^10^, C^30^, TE^30^, S^10^	*TEM, SHV, MCR*-1, *hep*	*ISS*
27	O55	2020	AMC^30^, AMP^10^, CTX^30^, C^30^, CIP^5^, NX^10^, NA^10^, TE^30^, S^10^	*CTX, TEM, SHV, MCR*-1, *hep*	*ISS*
28	O157	2020	AMC^30^, AMP^10^, C^30^, NA^10^, TE^30^, SXT, S^10^	*TEM, MCR*-1, *hep*	*ISS*

*E. coli=Escherichia coli*, AMC=Amoxicillin-clavulanate, AMP=Ampicillin, CIP=Ciprofloxacin, CTX=Cefotaxime, C=Chloramphenicol, CT=Colistin sulfate, IMP=Imipenem, NA=Nalidixic acid, NX=Norfloxacin, S=Streptomycin, SXT=Sulfamethoxazole-trimethoprim, T=Tetracycline

### Molecular characterization of several virulences and antimicrobial resistance genes of *E. coli* isolates

**Figure-3:**
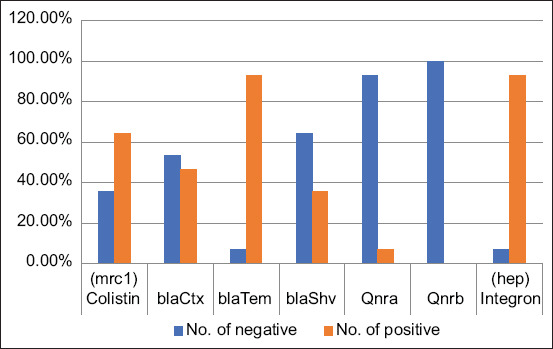
Percentage of the different antibiotic-resistant genes of isolated *Escherichia coli* strains.

Several resistance genes were identified in 28 phenotypically resistant *E. coli* isolates using PCR. A total of 26/28 *E. coli* isolates (93%) harbored one or more ESBLs. *Bla*TEM was found in 26 *E. coli* isolates (93%), followed by *bla*CTX-M and *bla*SHV (46.5% and 35.7%, respectively), as shown in Table-7 and Figure-3.

**Table-7:** The positive percentage of different examined antibiotic-resistant genes for isolated *E. coli* strains.

Results	*Colistin (mrc1)*	*bla*CTX-m	*bla*TEM	*bla*SHV	*Qnra*	*Qnrb*	*Integron (hep)*
No. of positive	18	13	26	10	2	0	26
Percentage (n = 28)	64.3	46.5	93	35.7	7	0	93

*E. coli=Escherichia coli*

In this study, two *E. coli* isolates harbored the *qnrA* gene while no isolate possessed the *qnrB* gene associated with quinolone resistance. The *mcr*-1 gene associated with CT resistance was detected in 18 *E. coli* isolates (Table-6). The correlation between the genotypic and phenotypic antimicrobial resistance of *E. coli* is shown in Table-6. A high prevalence of the *iss* gene (93%) was found, while that of the other virulence gene *papC* was much lower (32%).

## Discussion

*Escherichia coli* is one of the most widely distributed bacterial pathogens worldwide and causes severe economic losses in the poultry industry because of mortalities and costs expended on treatment. In addition, the emergence of MDR *E. coli* adds a further threat to the poultry industry because of the uncontrolled usage of antibiotics. Furthermore, MDR *E. coli* can spread to humans through food chains causing major public health dangers [47].

In total, *E. coli* was detected in 675/938 farms (72%). These results agreed with the previous report that demonstrated a high prevalence of *E. coli* in Egyptian poultry farms [2], where *E. coli* was isolated from 70% of chickens. Previous studies [48, 49] have also isolated *E. coli* in 34% and 26.7%, respectively, from chickens in Egypt. Conversely, in the results obtained here for 2017, the prevalence of *E. coli* was low (45.8%), which agrees with a prior report [50], which isolated *E. coli* from only 35% of chickens in Egypt. A higher isolation percentage of *E. coli* (88%) has been reported from poultry in the USA [19]. Furthermore, although the observed prevalence of *E. coli* in 2016 in our study was 98.3%, however previous studies [51, 52] reported *E. coli* isolation at very low percentages (13.4% and 11%) from poultry farms in Egypt and Ethiopia, respectively.

Our data showed that *E. coli* isolates could be serotyped into 13 serotypes, of which O125 (n = 8) and O111 (n = 5) were most prevalent. These results are similar to a previous study conducted by Badr *et al*. [53] but disagree with the prevalence of serotypes (O1, O2, O25, and O78) isolated in Jordan [54].

In our study, we tested *E. coli* strains for antimicrobial resistance against 18 different antibiotics. The highest antimicrobial resistance rates in this study ranged from 95% to 86.7% for AMP, P, NA, T, AMC + clavulanic acid, DOX, CTX, FOS, and trimethoprim. In contrast, the percentage of resistance for C, DFX, CIP, NX, LEV, ENR, S, and IMP ranged from 85.5% to 69.7%. The CT showed the lowest resistance percentage (49.7%). These phenotypic resistance rates have been previously reported [48, 49], although lower percentage rates for those phenotypic resistances have also been described [2, 51].

Most of the phenotypically antibiotic-resistant *E. coli* isolates harbor antibiotic resistance genes associated with resistance to colistin, β-lactams, and quinolones. The *mcr*-1 gene is associated with colistin resistance and is widely found in different bacteria belonging to *Enterobacteriaceae* isolated from various sources [55]. Although only one of *E. coli* isolates was phenotypically resistant to CT, the *mcr*-1 gene was detected in 18 (64.3%) of all tested *E. coli* isolates. The overall percentage of phenotypic resistance to CT reached 49.3%. This result agrees with a previous report [25], which reported *mcr*-1 gene detection at a prevalence of 41.83%. These findings suggest that poultry farms might be a source of colistin-resistant *E*. *coli*. However, this percentage was lower than in *E. coli* isolates from chicken meat samples (19.5%) [56] and 8% (8/100) of *E. coli* isolates from healthy broilers in Pakistan [57]. Therefore, the emergence and spread of colistin-resistant *E*. *coli* in animals and animal by-products, such as chicken meat, may become a serious public health problem as quinolones and β-lactamases are extensively used to treat many infectious diseases [58].

Extended-spectrum β-lactamases have a global distribution [59]. In this study, the overall resistance to CTX, a member of the β-lactamase group, was 86.7%. However, the detection of the genes responsible for antimicrobial resistance for the β-lactamase group (*bla*CTX-M, *bla*TEM, and *bla*SHV) was 46.5%, 93%, and 35.7%, respectively. Certainly, all the strains isolated here showed phenotypic resistance patterns to several antimicrobials related to β-lactamases. New ESBL-encoding genes (such as *bla*CTX-M, *bla*GES, or *bla*VEB-1) are usually located on integron-like structures [60].

Recent studies reported that a higher rate of integrons could lead to significant antibiotic resistance and, consequently, the emergence of ESBL and MDR isolates, which could be a serious risk to healthcare systems as well as the livestock and poultry industries [61, 62]. The *bla*TEM and *bla*SHV genes were detected in *E. coli* isolated from broilers suffering from septicemia in Egypt [63]. In this study, the *bla*TEM resistance gene was detected in 93% of *E. coli* isolates, which is lower than that detected in *E. coli* isolates from healthy broilers in Egypt (20.6%) [51]. Furthermore, a previous study revealed a high prevalence of integron 1 in *E. coli* isolates from different animal sources in Iraq [64].

Quinolones are mostly used for controlling infections, including those of Gram-negative bacteria such as *Enterobacteriaceae*. Fluoroquinolones have a broad-spectrum intrinsic activity that is greater than that of quinolones [65]. Plasmid quinolone resistance genes harbor many *qnr* alleles, which have been found on plasmids or bacterial chromosomes that are mainly associated with *Enterobacteriaceae* and are grouped into five distinct families: *qnrA*, *qnrB*, *qnrC*, *qnrD*, and *qnrS* [66, 67].

Resistance to the antimicrobials NX, CIP, LEV, and NZ, and the isolates were present in 73.2%, 73.5%, 69.7%, and 92.2% of the isolates, respectively. The same examined strains showed low percentages of 7% and 0% for *qnrA* and *qnrB*, respectively, but this may be because quinolone resistance is controlled by another group of genes, such as integrons, which were detected in 93% of isolates. These findings are lower than those described previously by Belotindos *et al*. [18], who detected the *Qnr* family (*qnrA1*, *qnrB4*, and *qnrS1*) in all tested isolates.

Integrons were detected in 93% of *E. coli* isolates in this study, which disagrees with a previous study by Moawad *et al*. [68], who did not record integrons in *E. coli* isolates from raw chicken samples in Egypt.

**Figure-4:**
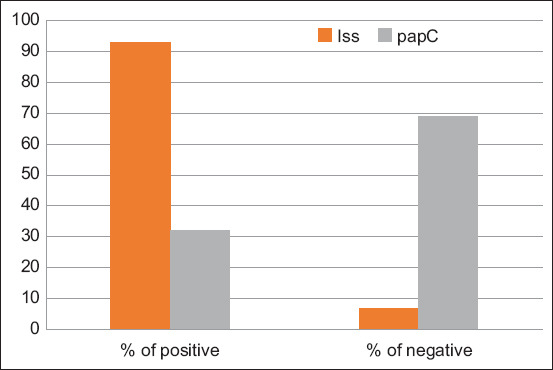
The percentage of the presence of the two examined virulence genes among *Escherichia coli* strains.

Avian pathogenic *E. coli* (APEC) isolates carry a wide range of virulence genes, such as adhesions, toxins, siderophores, iron transport systems, and invasions that increase pathogenicity in avian colibacilloses [50, 69]. Several virulence genes, including *papC*, are important in adherence [69]. A high percentage (93%) of the isolates contained the *iss* gene, as shown in Figure-4, although the *papC* gene was present at a lower percentage (32%). A previous study conducted by Sedeek *et al*. [70] reported that there is neither a uniform nor an absolute combination of the virulence genes that can distinguish between APEC and non-APEC strains of *E. coli*.

Furthermore, detecting the *iss, tsh*, and *papC* genes exclusively in the APEC strains could be consistent as important colibacillosis virulent factors [71, 72]. Our findings were similar to those previously reported by Johar *et al*. [73] for examination of different virulence genes from healthy and unhealthy chickens in Qatar, where the *iss* gene was more predominant in APEC in healthy birds (97%) than among unhealthy ones (16%).

In 2006, Avian Influenza outbreak occurred in Egypt, and consequently, all efforts were made to confront this at the expense of other diseases. Furthermore, a repeated problem is a lack of control and monitoring of indiscriminate use of antibiotics in treatment or as growth promoters to increase productivity, which all contribute to the high percentage of antimicrobial resistance [74].

## Conclusion

The high prevalence of *E. coli* in poultry farms in Egypt and the development of MDR *E. coli* are of considerable concern which has been developed from the uncontrolled usage of antimicrobials. Furthermore, the detection of different antibiotic resistance genes, such as colistin resistance, poses a significant threat to public health. Consequently, additional investigation and surveillance programs are required to focus on the development of antimicrobial resistance in the field, which facilitates its transmission to humans through the food chain. Improved regulatory control of administration of these antibiotics to avoid the generation of antibiotic-resistant strains is needed to protect public health.

## Authors’ Contributions

MAAA, HR, HB, EAH, MFA, HKS, and WMH: Designed the study, collected the samples, analyzed the data and conducted the bacterial isolation and the biochemical and antimicrobial susceptibility tests. AGS and AAM: Perform the PCR assays. MAAA, MAS, AGS, and EAH: Wrote the manuscript. All authors have read and approved the final manuscript.
